# The complete chloroplast genome sequence of *Pericampylus glaucus*

**DOI:** 10.1080/23802359.2019.1688718

**Published:** 2019-12-05

**Authors:** Hongmei Kang, Yi Wang

**Affiliations:** Laboratory of Forest Plant Cultivation and Utilization, Yunnan Academy of Forestry, Kunming, People’s Republic of China

**Keywords:** *Pericampylus glaucus*, chloroplast, Illumina sequencing, phylogenetic analysis

## Abstract

The first complete chloroplast genome (cpDNA) sequence of *Pericampylus glaucus* was determined from Illumina HiSeq pair-end sequencing data in this study. The cpDNA is 162,450 bp in length, contains a large single-copy region (LSC) of 90,871 bp and a small single-copy region (SSC) of 21,137 bp, which were separated by a pair of inverted repeats (IR) regions of 25,221 bp. The genome contains 130 genes, including 85 protein-coding genes, 8 ribosomal RNA genes, and 37 transfer RNA genes. The overall GC content of the whole genome is 38.0%, and the corresponding values of the LSC, SSC, and IR regions are 36.2, 32.1, and 43.5%, respectively. Further, phylogenomic analysis showed that *P. glaucus* and *Stephania japonica* clustered in a clade in family Menispermaceae.

*Pericampylus glaucus* is the species of the genus *Pericampylus* with the family of Menispermaceae. It is distributed in the south of China and Malaysia (Kifayatullah et al. 2017*). Pericampylus glaucus* is used as a traditional medicine in Asian for a long time. (Mahboob et al. 2017). It has pharmacological activities including anti-diabetic and anti-hyperlipidemic (Kifayatullah and Sengupta 2016). The extract of *P. glaucus* also has good properties against AIDS, HBV, and HCV virus (Zhao and Cui 2009; Kifayatullah et al. 2015). However, there have been no genomic studies on *P. glaucus.*

Herein, we reported and characterized the complete *P. glaucus* plastid genome (MN539265). One *P. glaucus* individual (specimen number: 5309270267) was collected from Cangyuan, Yunnan Province of China (23°17′5″ N, 99°5′49″ E). The specimen is stored at Yunnan Academy of Forestry Herbarium, Kunming, China and the accession number is YAFM20180328. DNA was extracted from its fresh leaves using DNA Plantzol Reagent (Invitrogen, Carlsbad, CA, USA).

Paired-end reads were sequenced by using Illumina HiSeq system (Illumina, San Diego, CA, USA). In total, about 21.1 million high-quality clean reads were generated with adaptors trimmed. Aligning, assembly, and annotation were conducted by CLC de novo assembler (CLC Bio, Aarhus, Denmark), BLAST, GeSeq (Tillich et al. 2017), and Geneious version 11.0.5 (Biomatters Ltd, Auckland, New Zealand). To confirm the phylogenetic position of *P. glaucus*, other three species of family *Menispermaceae* from NCBI were aligned using MAFFT version 7 (Katoh and Standley 2013). The Auto algorithm in the MAFFT alignment software was used to align the eight complete genome sequences and the G-INS-i algorithm was used to align the partial complex sequecnces and maximum likelihood (ML) bootstrap analysis was conducted using RAxML (Stamatakis 2006); bootstrap probability values were calculated from 1000 replicates. *Berberis koreana* (KM057375) and *Ranzania japonica* (MG234280) were served as the out-group.

The complete *P. glaucus* plastid genome is a circular DNA molecule with the length of 162,450 bp, contains a large single-copy region (LSC) of 90,871 bp and a small single-copy region (SSC) of 21,137 bp, which were separated by a pair of inverted repeats (IR) regions of 25,221 bp. The overall GC content of the whole genome is 38.0%, and the corresponding values of the LSC, SSC, and IR regions are 36.2, 32.1, and 43.5%, respectively. The plastid genome contained 130 genes, including 85 protein-coding genes, 8 ribosomal RNA genes, and 37 transfer RNA genes. Phylogenetic analysis showed that *P. glaucus* and *Stephania japonica* clustered in a unique clade in family *Menispermaceae* (Figure 1). The determination of the complete plastid genome sequences provided new molecular data to illuminate the *Menispermaceae* evolution.

**Figure 1.**
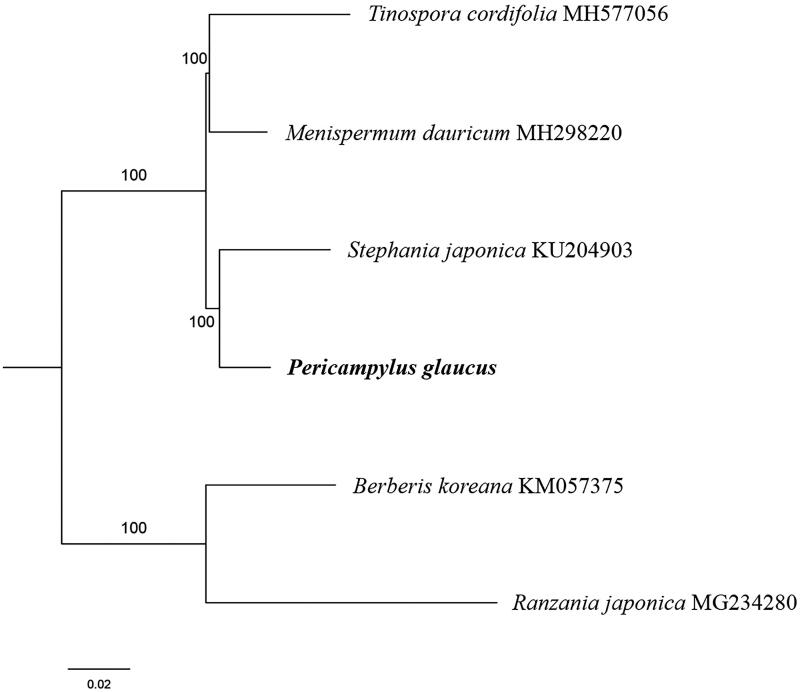
The maximum-likelihood tree based on the four chloroplast genomes of *Menispermaceae*. The bootstrap value based on 1000 replicates is shown on each node.
